# Chemical Composition, an Antioxidant, Cytotoxic and Microbiological Activity of the Essential Oil from the Leaves of *Aeollanthus suaveolens* Mart. ex Spreng

**DOI:** 10.1371/journal.pone.0166684

**Published:** 2016-12-01

**Authors:** Rosany Lopes Martins, Ranggel Carvalho Simões, Érica de Menezes Rabelo, Ana Luzia Ferreira Farias, Alex Bruno Lobato Rodrigues, Ryan da Silva Ramos, João Batista Fernandes, Lourivaldo da Silva Santos, Sheylla Susan Moreira da Silva de Almeida

**Affiliations:** 1 Laboratory of Pharmacognosy and Phytochemistry-Federal University of Amapá, Macapá-Amapá, Brazil; 2 Department of Chemical, Federal University of São Carlos, São Carlos – São Paulo – Brazil; 3 Laboratory of Chemistry, Federal University of Pará, Belém – Pará – Brasil; National Taiwan University, TAIWAN

## Abstract

*Aeollanthus suaveolens* species popularly known as catinga de mulata belongs to the Lamiaceae family. In the Amazon region, it is used in folk medicine for the treatment of gastritis, convulsions of epileptic origin, stomach pain and diarrhea in the form of tea and juice. Essential oils have analgesic, anti-inflammatory, and antimicrobial activity. This study evaluated the chemical composition of the *A*. *suaveolens* essential oil, and its cytotoxic, antimicrobial and antioxidant activity on *Artemia salina* Leach. The plant species was collected in Fazendinha district in the city of Macapa-AP. The essential oil obtained from the process was performed by hydrodistillation and identification of components by gas chromatography coupled with mass spectrometer. The antioxidant activity was evaluated by the kidnapping method of 2,2- diphenyl -1-picrilhidrazil radical, while the cytotoxic activity was assessed using saline A. and the microbiological activity was carried out by microdilution method with *Escherichia coli*, *Salmonella* sp. and *Staphylococcus aureus* bacteria. In a chromatographic analysis, the major constituents found in the essential oil of *A*. *suaveolens* were (E) -β-farnesene (37.615%), Linalool (33.375%), α-Santalene (3.255%) and linalyl acetate (3.222%). The results showed that the *Escherichia coli* and *Salmonella* sp. bacteria were more susceptible to MIC 50 mg.mL^-1^ when compared with the *Staphylococcus aureus* bacterium MIC 100 mg.mL^-1^. With respect to MBC concentration of 100 mg.mL^-1^ it was sufficient to inhibit the growth of *E*. *coli*. The essential oil did not show antioxidant activity, however, has a high cytotoxic activity against the *A*. *salina*, LC_50_ 8.90 μg.mL^-1^.

## Introduction

The use of natural products as raw material for synthesis of bioactive substances, especially drugs, has been widely reported over the time [1]. It is estimated that approximately 80% of the world often employs indigenous or traditional medicines for their primary health care needs, especially those that use therapies that involve the use of herbal medicines. Brazil has a huge biodiversity, where much of the native plant species has not been studied. It is estimated that about 3000 essential oils are known, of which about three hundred are commercially important, intended mainly for the fragrance market [2, 3].

Essential oils are organic compounds of heterogeneous chemical structure that are widely distributed in higher plants. The constituents of the essential oils may belong to several classes of compounds, but terpenes and phenylpropenes are the most commonly compounds found. Terpenes found more frequently in essential oils are monoterpenes, sesquiterpenes, and diterpenes minor constituents of essential oils [4].

The Lamiaceae family comprises 240 genders and 7200 species of nearly cosmopolitan distribution. In Brazil, there are 46 native genera and about 524 species [5]. In folk medicine, the Lamiaceae family ranks third in order of importance, with many species with biologically active substances [6].

The species *A*. *suaveolens* popularly known as catinga de mulata, belongs to the Lamiaceae family. It is an herb with about 40 cm tall, circular stem branched, petiolate leaves, coated with trichomes with aromatic essence in its pre-flowering and valve. It has metaclamideas flowers type bisexual, trimerous. Its androecium has didynamous stamens, spherical pollen grains, with supero and unilocular carpels. It has its ovarian in a gynobasic type supero, and the inflorescence racemes [7].

In folk medicine, *A*. *suaveolens* is used to control epileptic seizures, combat fever, headache, the onset of stroke, "quebranto" (it is a kind of bad enchantment). Its sheet is utilized in form of tea and juice [7]. In riverine community of the Solimões River in Amazonas, the species is used to treat gastritis, stomach pain, and diarrhea. Their baths are used to relieve symptoms in victims of "mau-olhado" (it is also a kind of bad enchantment) [8].

Few studies have been reported on a cytotoxic, antimicrobial and antioxidant activity of essential oil species of this family. Therefore, this study aimed to investigate the chemical composition, antimicrobial activity (against gram-negative and gram-positive), toxicity (compared to the larvae of *Artemia salina*), and antioxidant activity of the essential oil obtained from the leaves of *A*. *suaveolens*.

## Materials and Methods

### Plant material

The leaves of *A*. *suaveolens* were collected from Fazendinha district (00° 02'23 "S and 51° 06'29" W) in the Municipality of Macapa, Amapá. Two samples of plant species have been deposited in the Herbarium Amapaense (HAMAB) of the Institute of Scientific Research and Technology of the State of Amapá (IEPA) under the registration No M.R.L. 001. The study was conducted on private land with the permission and consent of the owner to carry out the study on site.

### Essential oil obtaining

The essential oil (EO) was obtained by hydrodistillation process using the Clevenger type apparatus, using 30 g of dried and crushed leaves of *A*. *suaveolens* for a period of 2 h at a temperature of approximately 100°C [9]. The essential oil was removed with the aid of a pasteur pipette, and then packaged in the amber bottle wrapped in aluminum foil and kept under refrigeration (4°C) for later analysis.

### Essential oil constituents of identification

The identification of essential oil components was performed by gas chromatography coupled to mass spectrometry (GC-MS) using Shimadzu equipment, MEGCs QP-2010 Plus model. Column DB-5 HT, Mark J & W Scientific, 30 m long, 0.32 mm diameter, 0.10 micron Indicated film thickness, and nitrogen as carrier gas. The gas chromatograph operating conditions were: an Internal pressure of 56.7 kPa column, split ratio 1:20, the column gas flow of 1.0 mL / min. (210°C), injector temperature 220°C, the temperature detector or the interface (GC-MS) 240°C. The initial column temperature was 60°C, followed by an increase of 3°C/min. up to 240°C and maintained constant for 30 min. The mass spectrometer was programmed to perform readings in a range of 29–400 Da 0.5s, intervals with ionization energy of 70 eV. It was injected 1μL of each sample at a concentration of 10,000 ppm dissolved in hexane. The identification of the components was based on the comparison of their Kovats index (KI) and mass spectra of each substance with the literature data.

### Antioxidant activity

The quantitative evaluation of antioxidant activity was based on the methodology proposed by Sousa et al. [10], Lopes-Lutz et al. [11] and Andrade et al. [12] before the use of 2,2-diphenyl-1-picryl-hidrazila (DPPH) with some adjustments to laboratory conditions.

A methanolic solution of DPPH was prepared (stock solution) at a concentration of 40 μg.mL^-1^, which was kept under the light. The essential oils were diluted in methanol at concentrations of 5, 2.5, 1, 0.75, 0.50 and 0.25 mg.mL^-1^. For the evaluation, there were added into a test tube 2.7 mL of the stock solution of DPPH, followed by addition of 0.3 mL of the essential oil solution. The white of the solution was prepared, this being a mixture of 2.7 mL methanol and 0.3 mL of a methanolic solution of each EO concentration evaluated. After 30 min. on a spectrophotometer (Biospectro SP-22), readings were performed at a wavelength of 517 nm [13]. The assay was performed in triplicate and the calculation of percentage of antioxidant activity (% AA) was calculated with the following equation:

$$(\%AA)=100-\left\{\frac{\left[\left({Abs}_{sample}-{Abs}_{white}\right).100\right]}{{Abs}_{control}}\right\}$$

%AA—percentage of antioxidant activity

Abs_sample_—Absorbance sample

Abs_white_—White Absorbance

Abs_control_—Control Absorbance

### Cytotoxic activity with *Artemia salina* leachg

The cytotoxicity assay against *A*. *salina* Leach was based on the technique of Araujo et al. [14] and Lobo et al. [15], with adaptations. an aqueous solution of synthetic sea salt was prepared (35.5 gL^-1^) for the incubation of 25 mg of *A*. *salina* eggs, which were placed in the dark for 24 h to larvae hatching (nauplius), then the nauplius were exposed to artificial light in 24 hour period to reach metanauplius stage. The mother solution was prepared to contain 54 mg of essential oil added 22.5 mL of synthetic sea salt solution and 4.5 mL of 5% dimethylsulfoxide (DMSO) to facilitate solubilization of it.

The metanauplius were selected and divided into 7 groups of 10 subjects in each test tube, held in triplicate. Each group received aliquots of the stock solution (2500, 1250, 625, 250, 25 and 2.5 uL), which were then completed to 5 mL volume with a synthetic sea salt solution to yield final solutions with the following concentrations 1000, 500, 250, 100, 10 and 1 μg.mL^-1^. Tests were performed in triplicates. To the test control, it was used a saline solution. After 24 hours it was counted the number of dead. The lethal concentration that causes 50% mortality in the population (LC_50_) was determined by probit analysis using the software SPSS [version 20.0; SPSS Inc., Chicago, IL, USA].

### Antimicrobial activity

#### Microorganisms

The evaluation of the antimicrobial activity of essential oil obtained from the leaves of *A*. *suaveolens* was tested against two gram-negative bacteria (*Escherichia coli* ATCC25922, *Salmonella sp*. ATCC14028) and two gram-positive bacteria (*Staphylococcus aureus* ATCC6538). Microorganisms were provided by the Oswaldo Cruz Foundation (FIOCRUZ). The bacteria were initially reactivated from stock cultures, maintained on Mueller-Hinton broth (MHC) for 18 h at 37°C.

#### Determination of the minimum inhibitory concentration (mic) and minimum bactericidal concentration (mbc)

The MIC determination was performed by diluting technical microplate (96 wells) according to the protocol established by the Clinical and Laboratory Standards Institute [16], with some adjustments.

The bacteria were initially taken from the stock cultures maintained on Mueller-Hinton broth (MHC) for 18 h at 37°C. After bacterial growth, an inoculum in 0.9% saline solution was prepared for each colony, adjusted to 0.5 McFarland scale, further diluted in MHC and tested in concentration 1,5 x10^7^ Colony forming unit (CFU/mL).

To determine the MIC, the EO was diluted in dimethylsulfoxide (4% DMSO). The orifices of the microplate were filled with 50 μL NaCl and 50 μL of solution OE *A*. *suaveolens*. Then serial dilutions were made from 100 to 0.048 μg.mL^-1^. Additionally, 50 μL was distributed suspensions of microorganisms in each well of the microplate. As a positive control, it was used amoxicillin (50 μL.mL^-1^). There were performed the control of the culture medium, EO control and the negative control (4% DMSO). The microplates were incubated at 37°C for 24h. The experiments were performed in triplicate. The MIC was considered the lowest concentration of EO which was not displayed microbial growth. The presence of turbidity in the wells indicated microbial growth and therefore there was no antimicrobial activity.

Determination MBC was performed based on the results obtained in testing the MIC. The wells of microplates of microbial growth were replicated in Muller-Hinton agar and incubated at 37°C for 24h. The MBC was considered as the lowest concentration of essential oil in which, there was no growth of microorganisms in the Petri dishes, and there was an elimination of microorganisms.

### Statistical analysis

The data analysis was performed by analysis of variance (ANOVA) and Tukey test to identify significant differences using BioEstat program [17]. The differences that showed lower levels of probability and equal to 5% (p = 0.05) were considered statistically significant. The results were expressed as mean ± standard deviation (SD).

## Results and Discussion

### Identification of chemical compounds by GC-MS of the *A*. *suaveolens* essential oil

The chemical composition of the essential oil from *A*. *suaveolens* was determined using GC-MS identification of major constituents present in this oil (Table 1) (S1 Fig). The chemical constituents detected in the oil extracted from the leaves, 1.57% are monoterpenes hydrocarbons, 39.65% are oxygenated monoterpenes, 43.56% are sesquiterpenes hydrocarbon, and 2.87% are lactones. As the major components (E) -β-farnesene (37.615%), linalool (33.375%), α-santalene (3.255%) and linalyl acetate (3.222%) (S2 Fig) (S3 Fig). These results corroborate the Tucker et al. [18] that link these compounds as the main major components of the essential oil of *A*. *suaveolens* [19].

**Table 1:** Chemical composition of the *Aeollanthus suaveolens* essential oil.

N°	t_R_ (min)	KI	Compounds	Relative Percentage (%)
1	7.163	979	β-pinene	0.411%
2	7.560	990	β-myrcene	0.263%
3	8.857	1029	limonene	0.127%
4	8.971	1031	1,8-Cineole	0.223%
5	9.566	1037	β –ocimene	0.768%
6	**11.648**	**1096**	**Linalol**	**33.275%**
7	14.267	1169	Borneol	0.178%
8	15.327	1188	α- Terpineol	1.569%
9	16.949	1229	Nerol	0.180%
10	18.088	1252	Geraniol	0.464%
11	18.168	1257	Linalyl acetate	3.222%
12	23.595	1298	Geraniol formate (CAS)	0.544%
13	25.049	1417	α-Santalene	3.255%
14	25.691	1434	(*E*)-α-Bergamoteno	1.805%
15	**26.680**	**1456**	**(*E*)-β-Farnesene**	**37.615%**
16	27.399	1472	Massoia lactona	2.496%
17	27.522	1505	(*Z*), (*E*)- α-farnesene	0.408%
18	27.678	1419	(E)–caryophyllene	0.475%
19	28.095	1494	δ- decalactone	0.375%
			Monoterpenes hydrocarbon	1.57%
			monoterpenes oxygenates	39.65%
			sesquiterpenes hydrocarbons	43.56%
			Lactone	2.87%
			**Total**	87.65

T R: retention time; KI *: Kovats Index

The chemical composition of essential oils varies according to edaphic factors of soil and weather the chemotype of species and by human action. However, a refined phytochemical screening of the EOs by GC-MS provide the chemical profile of the species, as well as contribute to the prediction of biological activities. According to Almeida et al. [20], linalool and the δ- decalactone has proven anticonvulsant activity, which may indicate a potential use of EOs of *A*. *suaveolens*.

### Essential antioxidant activity from *Aeollanthus suaveolens* essential oil by the DPPH radical capture method

The antioxidant capacity of natural products is related to its composition of phenolic compounds, and the effect of these is to interrupt the chain of free radicals in the initiation stage of the oxidative process [21].

The results obtained after the determination of the OE antioxidant activity at different concentrations are shown in Table 2. The OE reached its maximum antioxidant activity (43.5%) at a concentration of 5 mg.mL^-1^.

**Table 2:** Antioxidant activity of *Aeollanthus suaveolens* essential oil.

OE concentrations of *A*. *suaveolens* (mg.mL^-1^)	% AA
0.25	19.5±0.38^a^
0.50	21.5±0.09^b^
0.75	22.8±0.05^c^
1	24.1±0.44^d^
2.5	31,5±0.50^e^
5	43.5±0.33^f^

The values correspond to the mean and standard deviation of triplicates. Different letters indicate a significant difference (p <0.05). Equation antioxidant activity Y = 4,96x + 18.92.

The correlation between antioxidant activity (%) and the concentration of the OE showed a high IC_50_ value of 6.26 mg.mL^-1^, when compared with the standards, vitamin C and flavonoids rutin, with IC_50_ of 6.13 μg.mL^-1^ 6.71 μg.mL^-1^ and respectively [22]. According to Nascimento et al. [23], the greater the consumption of DPPH in a sample, the lower its IC _50_ and the greater its antioxidant activity.

For Beatović et al. [24], the OE antioxidant capacity is related to their major compounds, but this study has not observed the antioxidant activity. Studies with *Ocimun basilicum* species, and linalool as major compound showed strong antioxidant capacity [25]. On the other hand, Lu and Foo [26] believe that most compounds act synergistically together producing a broad spectrum of antioxidant properties and thus create an effective defense against free radicals.

### Toxicity on Artemia salina essential *Aeollanthus suaveolens* oil

The toxicity test on *A*. *salina* L. is widely used in bioassay due to be fast, reliable, and low cost. Furthermore, the *A*. *salina* toxicity, shows good correlation with antitumor activities [27], pesticide [28] and anti-*Trypanosoma cruzi* [29] for substances LC50 <1000 μg.mL^-1^.

Amarante [30] classified both organic extracts and aqueous extracts, as nontoxic because the LC_50_ is above 1000 μg.mL^-1^, with low toxicity if the LC_50_ exceeds 500 μg.mL^-1^, with slight toxicity is the LC_50_ 100 to 500 μg.mL^-1^ and very toxic to LC _50_ is less than 100 μg.mL^-1^.

Table 3 shows the mean mortality readings held in the 24 hour period the cytotoxic activity of essential oil from *A*. *suaveolens* sheet. The results showed that the essential oil from *A*. *suaveolens* showed high toxicity against the *A*. *salina*, LC_50_ 8.90 μg.mL^-1^ (Table 3) and correlation coefficient (R^2^ 0.93), p <0.0000.

**Table 3:** Essential oil toxicity of *Aeollanthus suaveolens* front of the larvae of *A*. *salina*.

Concentrations (μg.mL^-1^)	Mortality%
DMSO (negative control)	0±0,00^a,^
1	0±0,00^a, b^
10	76.76±0,00^c^
50	100±0,00^c^
100	100±0,00^c^
250	100±0,00^c^
500	100±0,00^c^
1000	100±0,00^c^

The values correspond to the mean and standard deviation of triplicates. Different letters indicate a significant difference (p <0.05).

There is not report in the literature about cytotoxic activity of the essential oil of A. suaveolens front of A. *salina*. However, the high toxicity observed (8.90 μg.mL^-1^) can be related to the synergism between monoterpenes and sesquiterpenes, present in the essential oil from *A*. *suaveolens*. Studies pro Boscardin et al. [31] on cytotoxic activity of volatile oil *Eucalyptus benthamii* in four tumor lines showed that the essential oil showed better results than the single compounds (α-pinene and γ-terpinene) to Jurkat, HeLa, and B16F10 cells.

Burt [32] believes that the mechanism of action of monoterpene and sesquiterpene compounds can be related to the displacement of these compounds toward the aqueous phase to the membrane structure causing toxic effects on both the structure and function of cell membranes.

### Microbiological activity of the essential *Aeollanthus suaveolens* oil

The discovery of new natural products with antibiotic potential is of considerable interest due to the growing resistance of many bacteria to antibiotics currently used for the treatment of infections [33].

Table 4 refers to a microbial activity of essential oil from A. suaveolens. The results showed that gram-negative bacteria *Escherichia coli* and *Salmonella* were more susceptible to the essential oil from *A*. *suaveolens*, with MIC 50 mg.mL^-1^ when compared with the gram-positive bacteria *Staphylococcus aureus* MIC 100 mg.mL^-1^. With respect to MBC, the OE concentration equivalent to 100 *A*. *suaveolens* mg.mL^-1^ was sufficient to inhibit the growth of *E*. *coli*.

**Table 4:** Minimum Inhibitory Concentration (MIC) and Minimum Bactericidal Concentration (MBC) of the essential oil *Aeollanthus suaveolens*.

Microorganism	MIC (mg.mL^-1^)	MBC (mg.mL^-1^)
*Escherichia coli*	50	100
*Staphylococcus aureus*	100	ND
*Salmonella sp*	50	ND
Amoxilina (Controle positivo)	0,048	0,048
DMSO (Controle negativo)	-	-

ND: Not Detected

Studies by Simionatto et al. [34] with isolated compounds from *A*. *suaveolens* analyzed by bioautography showed that the lactone monoterpene massoialactona exhibited excellent antibacterial property to *Salmonella Setubal* and *Bacillus subtilis* microorganisms, being active in the minimum concentration that was tested (3.125 μg.mL^-1^). In this study, however, the *A*. *suaveolens* EO showed better bacteriostatic activity (50 mg.mL^-1^) for *E*. *coli* and *Salmonella sp*. and bactericidal activity (100 mg.mL^-1^) only for *E*. *coli*. This may be due to the low yield of massoialactona (2.496%) compared with other compounds identified in this study.

The lipophilic character of monoterpene compounds and sesquiterpene present in this oil can explain the mechanism of action of antimicrobial activity of *A*. *suaveolens* in the essential oil. Since these compounds can cause damage to the cell membrane which subsequently affects the balance and homeostasis of pH and inorganic ions [35, 36].

Unlike what was observed in the EO, the cytotoxic activity of *A*. *suaveolens*, where the synergistic effect of monoterpene compounds and sesquiterpene appears to potentiate the toxicity against the larvae of *A*. *salina*, the antimicrobial isolates proved to be the most isolated active compounds (massoialactona) of all the essential oil components [34]. However, it is important to note that synergistic or antagonistic effects should not be excluded from antimicrobial activity [37].

## Conclusion

The essential oil obtained from the leaves of *Aeollanthus suaveolens* were chemically analyzed and determined the antioxidant, cytotoxic and antimicrobial activity. With regard to chemical analysis, the major compounds identified were (E) -β-farnesene, Linalool, α-Santalene, and linalyl acetate. The antimicrobial activity showed that the essential oil from *A*. *suaveolens* showed better bacteriostatic potential for gram-negative bacteria (*Escherichia coli* and *Salmonella*) than to gram-positive bacteria (*Staphylococcus aureus*) and potential bactericidal for gram-negative bacteria (*E*. *coli*). The essential oil showed little antioxidant activity by DPPH radical capture method when compared with standard vitamin C and rutin. However, it showed high Cytotoxic activity on *Artemia salina*. The data show the importance of preliminary bioassays as a screening of the biological potential of plant products, as well as the importance of these products as a source of bioactive compounds.

## Supporting Information

S1 Fig Chromatogram obtained by CG of essential *A*. *suaveolens* oil. (DOCX)

S2 Fig Phytochemical Profile of EO from *A*. *suaveolens*. (DOCX)

S3 Fig Spectrum of mass from essential *Aeollanthus suaveolens* oil, obtained by GC-MS in comparison with equipment library spectrum. (DOCX)
