# Supplementary material for: Chemical Composition, an Antioxidant, Cytotoxic and Microbiological Activity of the Essential Oil from the Leaves of Aeollanthus suaveolens Mart. ex Spreng
Source: PLoS One. 2016 Dec 1;11(12):e0166684. doi: 10.1371/journal.pone.0166684 (PMC5132230; doi:10.1371/journal.pone.0166684)
Supplement: S1 Fig — (DOCX) [file pone.0166684.s001.docx]

**SUPPORT MATERIAL**

**S1 Fig.** Chromatogram obtained by CG of essential *A. suaveolens* oil.


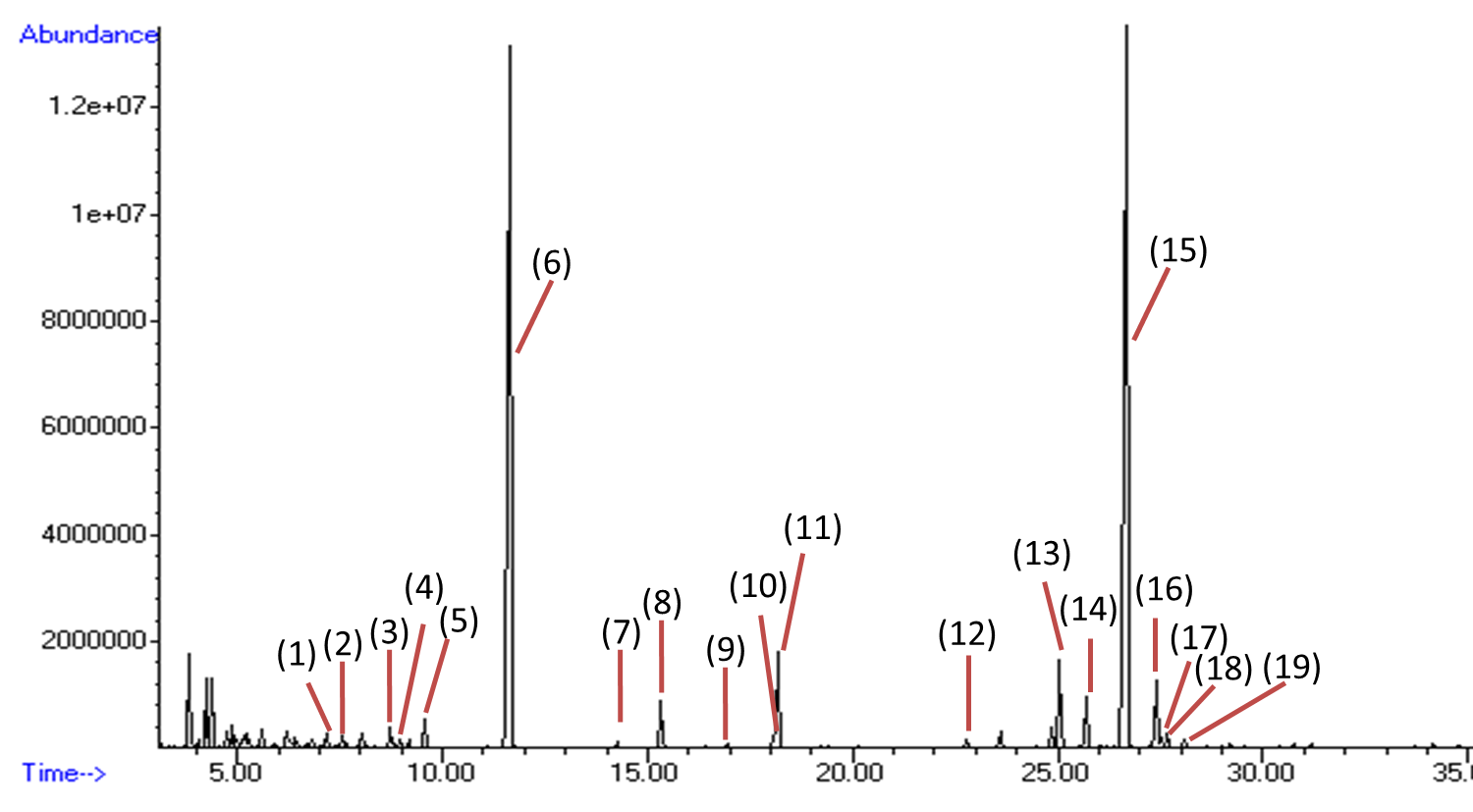


Conditions: Drag gas: Helium (He); initial temperature 60 ° C; initial time 1.0 min .; the column temperature increased 3 ° C / min. to 240 ° C, kept at this temperature for 30.0 min.
