# Supplementary material for: Chemical Composition, an Antioxidant, Cytotoxic and Microbiological Activity of the Essential Oil from the Leaves of Aeollanthus suaveolens Mart. ex Spreng
Source: PLoS One. 2016 Dec 1;11(12):e0166684. doi: 10.1371/journal.pone.0166684 (PMC5132230; doi:10.1371/journal.pone.0166684)
Supplement: S2 Fig — (DOCX) [file pone.0166684.s002.docx]

**S2 Fig.** Phytochemical Profile of EO from *A. suaveolens*

 The numbers of molecules are identified for each compound.
