# Supplementary material for: Chemical Composition, an Antioxidant, Cytotoxic and Microbiological Activity of the Essential Oil from the Leaves of Aeollanthus suaveolens Mart. ex Spreng
Source: PLoS One. 2016 Dec 1;11(12):e0166684. doi: 10.1371/journal.pone.0166684 (PMC5132230; doi:10.1371/journal.pone.0166684)
Supplement: S3 Fig — (DOCX) [file pone.0166684.s003.docx]

**S3 Fig.** Spectrum of mass from essential *Aeollanthus suaveolens* oil, obtained by GC-MS in comparison with equipment library spectrum.

**Substance (1)** - β-pinene (tR = 7.163 min.)

Library Mass spectrum

**Substance (2) -** β-myrcene (tR = 7.560 min.)

Library Mass spectrum

**Substance (3)** - Limonene (tR = 8857 min).

Library Mass spectrum

**Substance (4)** - 1,8-cineole (tR = 8971 min).

Library Mass spectrum

**Substance (5)** - Β - Ocimene (tR = 9566 min).

Library Mass spectrum

**Substance (6)** - L-Linalool (tR = 11 648 min.)

Library Mass spectrum

**Substance (7)** - Borneol L (Rt = 14.267min.)

Library Mass spectrum

**Substance (8)** - α- terpineol (tR = 15,327 min.)

Library Mass spectrum

**Substance (9)** - Nerol (tR = 16.949 min).

Library Mass spectrum

**Substance (10)** - (E) -Geraniol (tR = 18,088 min.)

Library Mass spectrum

**Substance (11)** - Linalyl acetate (tR = 18,168 min.)

Library Mass spectrum

**Substance (12)** - Geraniol format (CAS) (tR = 23.595 min.)

Library Mass spectrum

**Substance (13)** - α-santalene (tR = 25 049 min.)

Library Mass spectrum

**Substance (14)** - (*E*)-α-Bergamoteno (t_R_ = 25.691 min.)

Library Mass spectrum

**Substance (15)** - (*E*)-β-Farnesene (t_R_ = 26.680 min.)

Library Mass spectrum

**Substance (16)** - Massoia lactone (t_R_ = 27.399 min.)

Library Mass spectrum

**Substance (17)** - (*Z*), (*E*)- α-farnesene (tR = 27.522 min.)

Library Mass spectrum

**Substance (18)** - (*E*)- Caryophyllene (t_R_ = 27.678 min.)

Library Mass spectrum

**Substance (19)** - δ- Decalactone (t_R_ = 28.095 min.)

Library Mass spectrum
